# Unsterile Subcutaneous Insulin Injections Causing Psoas and Spinal Epidural Abscesses

**DOI:** 10.7759/cureus.18715

**Published:** 2021-10-12

**Authors:** Ebubechukwu Ezeh, Esiemoghie J Akhigbe, Joseph Simmons, Mohamed Suliman, Yousef Shweihat

**Affiliations:** 1 Internal Medicine, Marshall University Joan C. Edwards School of Medicine, Huntington, USA; 2 Cardiology, Marshall University Joan C. Edwards School of Medicine, Huntington, USA; 3 Pulmonary and Critical Care, Marshall University Joan C. Edwards School of Medicine, Huntington, USA

**Keywords:** spinal abscess, epidural abscess, unsterile insulin needles, spinal compression, mrsa bacteremia

## Abstract

Spinal epidural and psoas abscesses have been found to occur together. Most cases described in the literature have been secondary to either hematogenous spread or direct invasion. Risk factors include intravenous drug use and immunosuppression. This case highlights the risk of the use of unsterile subcutaneous insulin injections leading to psoas abscess, which can be complicated by a spinal epidural abscess.

## Introduction

Most patients with spinal epidural and/or psoas abscess have one or more predisposing conditions, such as an underlying disease diabetes mellitus, alcoholism, renal disease, infection with human immunodeficiency virus, or other forms of immunosuppression [1]. They have also been reported after interventions like surgery and after epidural anesthesia [2,3]. We present a case of a 64-year-old male who developed psoas and epidural abscesses from unsterile subcutaneous insulin injections.

## Case presentation

**Figure 1:**
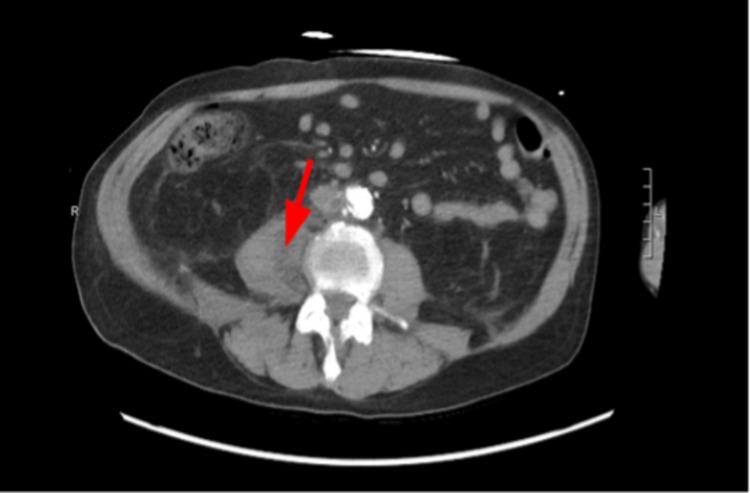
Initial CT showing psoas abscess (red arrow).

A 64-year-old male with a past medical history significant for insulin-dependent diabetes mellitus and substance abuse presented after being found lying on the floor. He was confused and as such history was limited at the time. He had back pain with lower extremity weakness. He lived alone but was last seen normal a day prior to the presentation. The patient also mentions recent tick bites. He was tachycardic with a heart rate of 105 beats/minute and a fever of 101 °F. Notable examination findings included reduced power across lower extremities and increased tone across knee and ankle joints. Heart sounds one and two were auscultated; murmurs were not appreciated. Notable laboratory findings included white blood cell counts 14.5 × 10^3^ cells per cubic millimeter with 17% bands, creatine kinase 16,000 units/liter, lactate 3 millimole/liter, tick serology was positive for Rocky Mountain spotted fever, and blood culture that was positive for methicillin-resistant *Staphylococcus aureus* (MRSA). CT scan of the lumbar spine showed spondylodiscitis at L4-L5 with epidural and right psoas intramuscular abscesses as shown in Figure 1.

**Figure 2:**
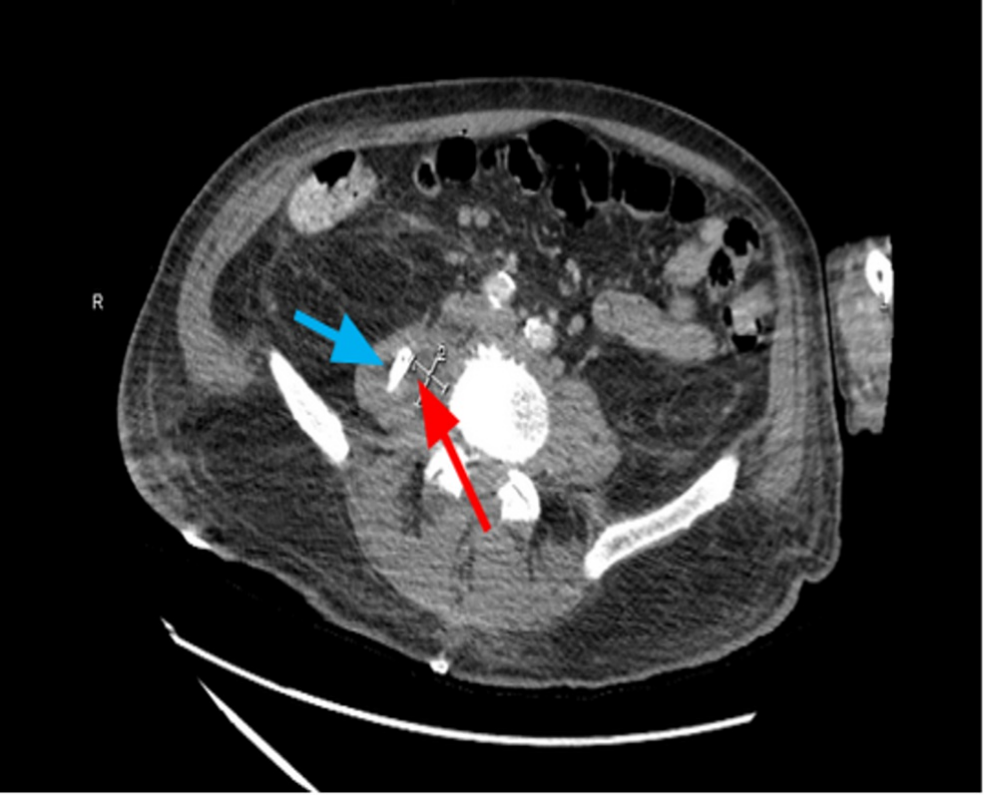
Abdominal CT scan with IV contrast showing persistent fluid collection (red arrow) despite CT-guided placement of a pigtail catheter (blue arrow).

He was started on intravenous antibiotics that included vancomycin, ceftriaxone, and doxycycline. The neurosurgery team performed a decompressive laminectomy of epidural abscess. Follow-up CT abdomen/pelvis, however, showed that the psoas abscess was unchanged from the original size. Psoas abscess drainage was performed by interventional radiology (IR). But patient continued to have spikes of fever of up to 102 °F and blood cultures continued to grow MRSA. Repeat imaging at this time showed expanding right psoas abscess as shown in Figure 2. This necessitated additional CT-guided drainage.

**Figure 3:**
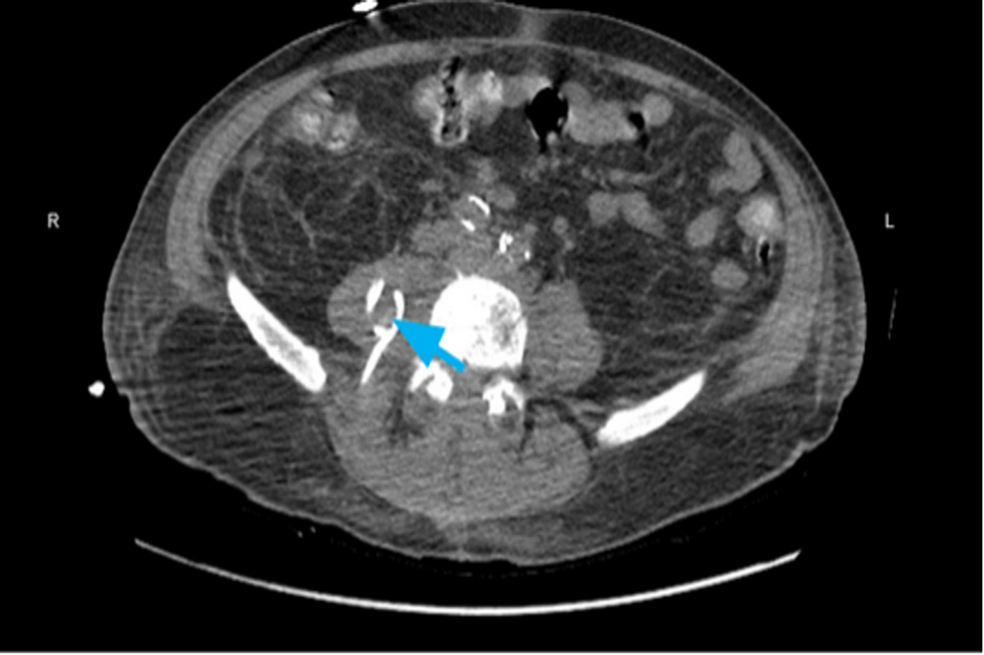
CT abdomen without contrast showing resolution of the fluid collection within the right psoas muscle after placement of a second catheter (blue arrow).

It was at this point that post-procedure imaging showed collapsed right psoas abscess with no residual fluid accumulation (Figure 3).

The patient’s symptoms markedly improved; his labs normalized, and he remained afebrile. Upon further questioning at this time, the patient revealed that he was reusing his subcutaneous insulin needles and injecting them in the same location each time. This was suspected to be the most likely source of his MRSA bacteremia.

## Discussion

Patients with MRSA bacteremia should be evaluated for epidural and psoas abscesses. Epidural abscesses and psoas abscesses are interrelated infections, with one capable of leading to the other. They may result from a local contiguous source or from hematogenous dissemination [4]. In fact, about 10 to 30% of spinal epidural abscesses result from direct extension of local infections like psoas abscesses [3]. Both entities share the same risk factors of intravenous drug abuse, diabetes mellitus, and immunosuppression. Because most predisposing conditions allow for invasion by skin flora, *S. aureus* causes about two-thirds of cases [2]. This was the case with our patient who repeatedly injected insulin into the same spot while reusing needles. The most common causative organism for both spinal epidural and psoas abscesses is *S. aureus*, especially following hematogenous or contiguous extension from a non-intraabdominal source [5].

While epidural abscesses commonly present as a triad of fever, back pain, and neurologic deficits, psoas abscess usually presents as fever, lower abdominal or back pain and pain referred to the hip [6]. Imaging studies such as MRI and blood cultures are the mainstays of diagnosis. Our patient had MRSA bacteremia. Bacteremia causing or arising from a spinal epidural abscess is detected in about 60% of patients [7]. This is especially so in those infected with *S. aureus* like our patient than with other organisms [8]. Early antibiotic therapy and surgical drainage of abscesses are key to complete recovery.

In patients with epidural abscess, compression and ischemia are believed to have an additive adverse effect on neurologic function in patients [9]. The remarkable degree of neurologic improvement in some patients after decompressive laminectomy provides evidence of mechanical pathophysiology, but thrombosed levels are observed in few postmortem examinations [10].

## Conclusions

Patients with either psoas or spinal epidural abscess may need to be evaluated for the other, especially if symptoms persist. Also, this case emphasizes the value of complete history-taking inpatient care. It is important to obtain further history in patients with injectable medications when trying to identify other potential sources of an abscess/bacteremia. Patients should also be counseled on the proper disposal of single-use needles.
